# Acetylation-Mediated Suppression of Transcription-Independent Memory: Bidirectional Modulation of Memory by Acetylation

**DOI:** 10.1371/journal.pone.0045131

**Published:** 2012-09-19

**Authors:** Katja Merschbaecher, Jakob Haettig, Uli Mueller

**Affiliations:** 1 Dept. 8.3 Biosciences Zoology/Physiology-Neurobiology, ZHMB (Center of Human and Molecular Biology), Saarland University, Saarbrücken, Germany; 2 Department of Neurobiology and Behavior, Center for the Neurobiology of Learning and Memory, University of California Irvine, Irvine, California, United States of America; University of Missouri, United States of America

## Abstract

Learning induced changes in protein acetylation, mediated by histone acetyl transferases (HATs), and the antagonistic histone deacetylases (HDACs) play a critical role in memory formation. The status of histone acetylation affects the interaction between the transcription-complex and DNA and thus regulates transcription-dependent processes required for long-term memory (LTM). While the majority of studies report on the role of elevated acetylation in memory facilitation, we address the impact of both, increased and decreased acetylation on formation of appetitive olfactory memory in honeybees. We show that learning-induced changes in the acetylation of histone H3 at aminoacid-positions H3K9 and H3K18 exhibit distinct and different dynamics depending on the training strength. A strong training that induces LTM leads to an immediate increase in acetylation at H3K18 that stays elevated for hours. A weak training, not sufficient to trigger LTM, causes an initial increase in acetylation at H3K18, followed by a strong reduction in acetylation at H3K18 below the control group level. Acetylation at position H3K9 is not affected by associative conditioning, indicating specific learning-induced actions on the acetylation machinery. Elevating acetylation levels by blocking HDACs after conditioning leads to an improved memory. While memory after strong training is enhanced for at least 2 days, the enhancement after weak training is restricted to 1 day. Reducing acetylation levels by blocking HAT activity after strong training leads to a suppression of transcription-dependent LTM. The memory suppression is also observed in case of weak training, which does not require transcription processes. Thus, our findings demonstrate that acetylation-mediated processes act as bidirectional regulators of memory formation that facilitate or suppress memory independent of its transcription-requirement.

## Introduction

Long-term memory (LTM), and long-lasting synaptic changes are characterized by their dependence on protein synthesis and gene expression [1]–[3]. These changes in gene expression are induced by a series of conserved second messenger mediated events that finally change the activity of transcription factors, and thus gene expression [4]–[6]. While the majority of these studies focused on events regulated via phosphorylation, more recent studies point to an important role of protein acetylation in synaptic plasticity, and memory formation [7]–[9].

Acetylation of histone tails by histone acetyltransferases (HATs) leads to loosening of the histone-DNA interactions, enabling access of the transcription machinery [10], [11]. Work in *Aplysia* and rodents demonstrated that transcriptional co-activators like CBP (CREB binding protein), p300, and the p300/CBP associated factor (PCAF) have intrinsic HAT activities, essential for gene expression underlying long-lasting neuronal plasticity [12]–[17]. Studies using inhibitors of histone deacetylases (HDAC) support the facilitating role of elevated acetylation levels on transcription-dependent processes. In presence of HDAC inhibitors, sub-threshold stimulation, or a weak training, is sufficient to trigger long-term facilitation (LTF) in *Aplysia* [13], to facilitate memory formation in crabs [18], and to enhance long-term potentiation (LTP), or memory in rodents [19]–[24].

A study using *Aplysia* neurons demonstrates that excitatory and inhibitory inputs leading to activation, or suppression of gene expression involve different acetylation-dependent processes [13]. The balance between activation and suppression of gene expression plays a critical role in memory formation [4], and transcription efficiency is regulated by acetylation. Assuming that learning-induced changes in acetylation are bidirectional and depend on training strength we propose that weak training also induces a down-regulation of acetylation in order to prevent transcription-dependent processes. To test this hypothesis we used the associative appetitive olfactory learning in honeybees [25]–[27] to monitor changes in acetylation after weak and strong training. We measured acetylation on histone 3 at positions H3K9 and H3K18, which are acetylated by different HATs as demonstrated in mice and cell culture studies [28]–[30]. Moreover, we tested the impact of increased and decreased acetylation levels on memory after weak and strong training.

## Results

### Depending on training strength, associative learning induces different acetylation dynamics

We used appetitive olfactory conditioning of the proboscis extension response (PER) in honeybees [25], [26] to study the connection between training strength, learning-induced acetylation-dependent processes, and memory formation. In the honeybee, as in other species, defined training parameters trigger specific signaling processes and thus determine the characteristics of the memory induced [27], [31].

**Figure 1:**
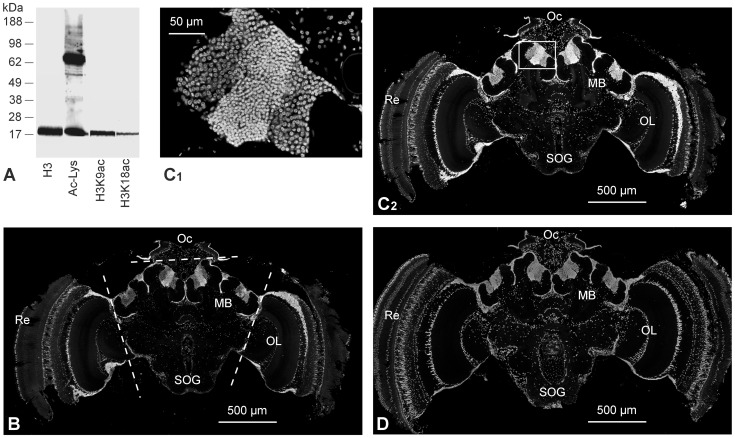
Characterization of antibodies used for quantification of protein acetylation in honeybee brain. (A) The antibodies against histone H3, H3K9ac, H3K18ac and acetylated lysine were tested on Western blots with separated protein from honeybee brain. All antibodies against H3 (and modifications) stain a single band at the molecular weight of H3. (B, C, D) Immunolabeling of the antigens recognized by antibodies against H3K9ac (B), H3K18ac (C) and H3 (D). The antibodies stain all somata in the honeybee brain. (C1) The higher magnification shows that labeling is restricted to the nuclei. Depicted are Kenyon cells of the mushroom bodies (MB). The brain area used for quantification of the acetylation status (dashed lines in B) contains somata from the central brain, antennal lobes, suboesophageal ganglia (SOB) (all together≈20%), and soma from Kenyon cells of the mushroom bodies (≈80%). The optical lobes (OL), the retina (Re) and the ocelli (Oc) were excluded from the measurements.

We first verified the specificity of the used antibodies in the honeybee brain by Western Blot. In honeybee brain tissue the antibodies against H3K9ac and H3K18ac each detect a single band with a molecular weight identical to that of histone H3 (Fig. 1A). We also tested a commercial anti-acetyl lysine antibody detecting a histone H3 corresponding band and several other bands of higher molecular weights. In immunohistochemistry of bee brain slices, the H3K9ac and H3K18ac antibodies selectively label the nuclei of neurons and glial cells (Fig. 1 B, C). Antibodies against H3 show the same selective labeling of nuclei (Fig. 1 D).

**Figure 2:**
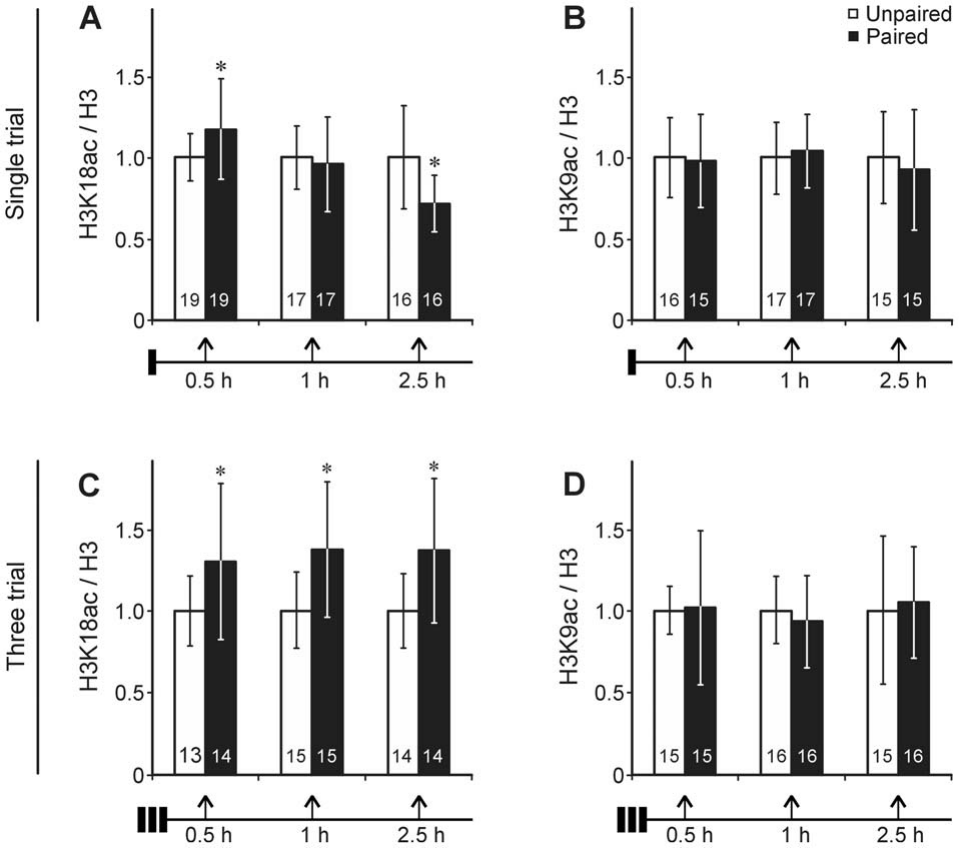
The dynamics in histone acetylation after appetitive associative conditioning depends on the training strength. In the paired groups honeybees either received a (A, B) single-trial conditioning (CS-US) or (C, D) a three-trial conditioning (3× CS-US with and ITI of 2 min), while in the unpaired groups CS and US was presented in unpaired order (US followed by CS after 15 s). At the indicated times (0.5, 1 and 2.5 hours) after training, the brains were dissected and the levels of acetylated histone H3K18 (H3K18ac) and acetylated histone H3K9 (H3K9ac) were quantified and related to the levels of H3 in each of the samples. Since the ratios of H3K9ac/H3 and H3K18ac/H3 of the unpaired stimulation at the times tested did not differ from each other, the ratios for each time point were normalized with respect to the unpaired control. The data represent the mean ± SD, the number of bees tested is indicated in each column. Asterisks indicate significant differences (Student's t-test (two-tailed); *p<0.05) (details in Results).

The antennal lobes and the mushroom bodies with their intrinsic neurons - the Kenyon cells - play an important role in olfactory learning and memory formation in insects [32], [33]. Hence we dissected and measured the central part of the brain as specified in Figure 1 B. In the dissected brain tissue the Kenyon cells represent >80% of the total number of somata [34]. We monitored learning-induced changes in acetylation status by ELISA technique, which allows for a highly accurate quantification. The honeybees received either a single-trial conditioning that leads to a memory that is insensitive to translation and transcription blockers and decays over days, or a three-trial conditioning that induces a stable translation and transcription-dependent LTM [27], [31], [35], [36]. The animals in the control groups received the US and the CS in an explicitly unpaired temporal pattern (US first and after 15 s CS). After conditioning the relative amounts of H3, H3K18ac and H3K9ac were determined in each of the brain samples at times indicated in Figure 2. As expected, the H3 signals did not differ between the different groups (Table 1) and thus are used for normalization of the H3K9ac and H3K18ac signals in each of the samples. Single- and three-trial conditioning cause significant changes in the relative H3K18 acetylation (H3K18ac/H3) but leave H3K9 acetylation (H3K9ac/H3) unaffected (Fig. 2). A single-trial conditioning induces an immediate increase in H3K18 acetylation (30 min) (t = 2.16; df = 25.7; p = 0.04) that decreases to a level below that of the unpaired control group at 2.5 h (t = 3.04; df = 23.1; p = 0.006) after training (Fig. 2A). In contrast, three-trial conditioning induces an increase of H3K18 acetylation (30 min) (t = 2.11; df = 18.4; p = 0.048) that stays elevated for at least 2 h (1 h: t = 2.91; df = 22.2; p = 0.008; 2 h: t = 2.66; df = 19.5; p = 0.015) (Fig. 2C).

**Table 1:** Associative conditioning does not affect the amount of H3.

Total H3	Single-trial conditioning		Three-trial conditioning	
	Unpaired	Paired	Unpaired	Paired
**0.5 h**	1.02±0.18 (19)	0.98±0.18 (19)	1.01±0.24 (15)	0.99±0.23 (15)
**1 h**	0.98±0.16 (17)	1.02±0.17 (17)	0.98±0.24 (16)	1.02±0.26 (16)
**2.5 h**	0.94±0.24 (16)	1.06±0.34 (16)	0.96±0.37 (15)	1.04±0.38 (16)

The values show the means ± SDs of the relative amount of H3 in the samples of the groups (unpaired and paired) after conditioning used for normalization of the data presented in Figure 2.

To allow for comparison, the different samples were measured on the same ELISA plates. ANOVA analysis of the different groups revealed no significant differences (all p values>0.9).

### Decreasing acetylation levels causes a suppression of memory, independent of the training strength

We used the HDAC inhibitor trichostatin A (TSA) and the HAT inhibitor Garcinol to manipulate protein acetylation in the honeybees. TSA is an inhibitor of HDAC class 1 and 2 only and does not inhibit class 3 (sirtuins) [37]. TSA has been widely used to elevate acetylation levels in studies on synaptic plasticity and learning [9]. Garcinol is an inhibitor of the HATs like p300 and p300/CBP associated factor (PCAF) [38]–[40] that so far has only been used once in a recent study to investigate the role of HATs in learning [41].

**Figure 3:**
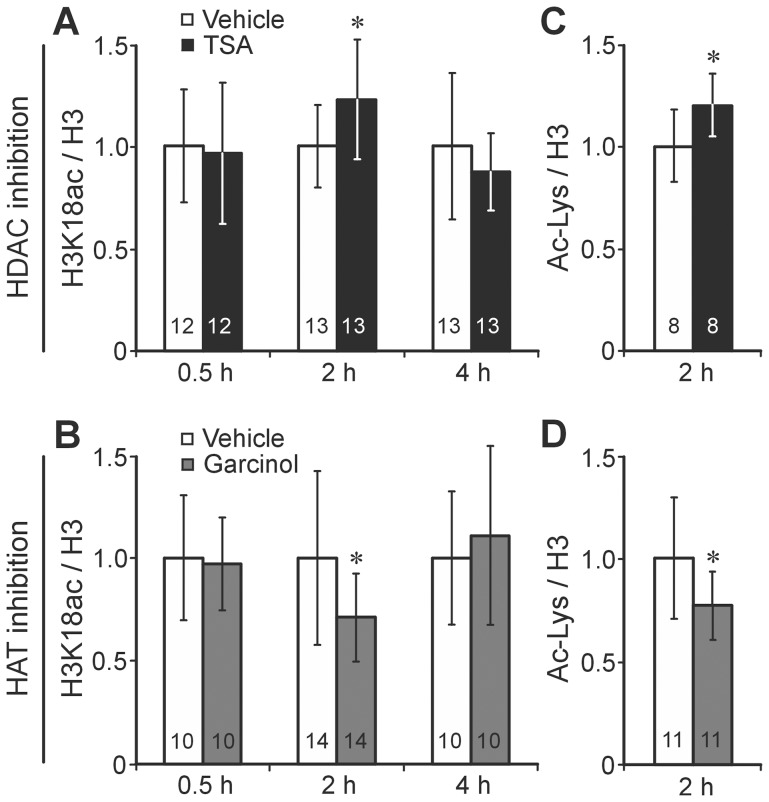
Injection of the HDAC inhibitor TSA or the HAT inhibitor Garcinol transiently increases or decreases protein acetylation in the honeybee brain. TSA (A, C) or Garcinol (B, D) and the corresponding vehicle were injected into the hemolymph of honeybees. At the indicated time (0.5, 2 and 4 h) after injection the brains were dissected and the levels of H3K18ac, H3K9ac and protein acetylation (Ac-K) were quantified and related to the levels of H3 in each of the samples. The data were normalized with respect to the corresponding vehicle control and represent the mean ± SD. The number of bees tested is indicated in each column. Asterisks indicate significant differences (Student's t-test (two tailed); *p<0.05) (details in Results).

We first determined the time after systemic injection at which the inhibitors TSA and Garcinol show their strongest effect on acetylation levels in the honeybee brain. As demonstrated with antibodies against histone H3, H3K18ac, and Ac-Lys the effects are at a maximum about 2 h after injection (Fig. 3). The inhibitory effect disappears about 4 h after injection. The signal measured by the antibody against H3 did not differ between the different groups (Table 2) and thus is used for normalization like in the previous experiment. As expected, the HDAC inhibitor TSA causes an increase, whereas the HAT inhibitor Garcinol causes a decrease in acetylation levels, both at the specific site H3K18 (2 h after TSA injection: t = 2.3; df = 21,3; p = 0.03; 2 h after Garcinol injection t = 2.3; df = 19.4; p = 0.03) (Fig. 3A, B). This effect is also observed in proteins other than histones (Ac-Lys: 2 h after TSA injection: t = 2.4; df = 11.2; p = 0.035; 2 h after Garcinol injection: t = 2.1; df = 15.8; p = 0.049) (Fig. 3 C, D). Before analyzing associative learning, we excluded the possibility that the used concentrations of TSA or Garcinol impair sensory processing of US (sucrose responsiveness) or non-associative forms of learning, such as sensitization and habituation (Table 3).

**Table 2:** TSA and Garcinol do not affect the amount of H3.

Total H3	HDAC inhibitor		HAT inhibitor	
	Vehicle	TSA	Vehicle	Garcinol
**0.5 h**	1.03±0.34 (12)	0.97±0.25 (12)	1.04±0.24 (10)	0.96±0.18 (10)
**2 h**	1.02±0.22 (13)	0.98±0.30 (13)	0.96±0.36 (14)	1.04±0.47 (14)
**4 h**	0.96±0.28 (13)	1.04±0.32 (13)	1.04±0.15 (10)	0.96±0.24 (10)

The values show the means ± SDs of the relative amount of H3 in the samples of the groups (vehicle and TSA or Garcinol) after injection used for normalization of the data presented in Figure 3. To allow for comparison, the different samples were measured on the same ELISA plates. ANOVA analysis of the different groups revealed no significant differences (all p values>0.9).

**Table 3:** HDAC inhibitor TSA and HAT inhibitor Garcinol neither affect gustatory responsiveness nor non-associative learning.

Behavioral test	HDAC inhibitor			HAT inhibitor		
	Vehicle	TSA		Vehicle	Garcinol	
**Responsiveness (PER)**	(23)	(24)		(28)	(27)	
0 mM sucrose	9%	4%	ns	4%	4%	ns
30 mM sucrose	22%	29%	ns	25%	27%	ns
1000 mM sucrose	100%	100%	ns	86%	89%	ns
**Sensitization (PER)**	(32)	(28)		(24)	(25)	
	19%	21%	ns	8%	4%	ns
**Habituation criterion**	(29)	(34)		(19)	(20)	
	24±13	23±12	ns	29±10	24±8	ns

Gustatory responsiveness, sensitization, or habituation of honeybees was tested 2 h after injection of TSA, or Garcinol, or the corresponding vehicle.

The data for responsiveness and sensitization show the percentage of animals that elicited the proboscis (PER). The habituation criterion presents the mean ± SD of responses until the bees show five successive failures to elicit a PER after repetitive sucrose stimulation to an antenna. The number (n) of tested bees is indicated in brackets. As revealed by Student's t-test (habituation) and Chi-square/Fisher exact test (responsiveness and sensitization) TSA and Garcinol both have no effect on the tested behavioral parameters (ns; p>0.6).

**Figure 4:**
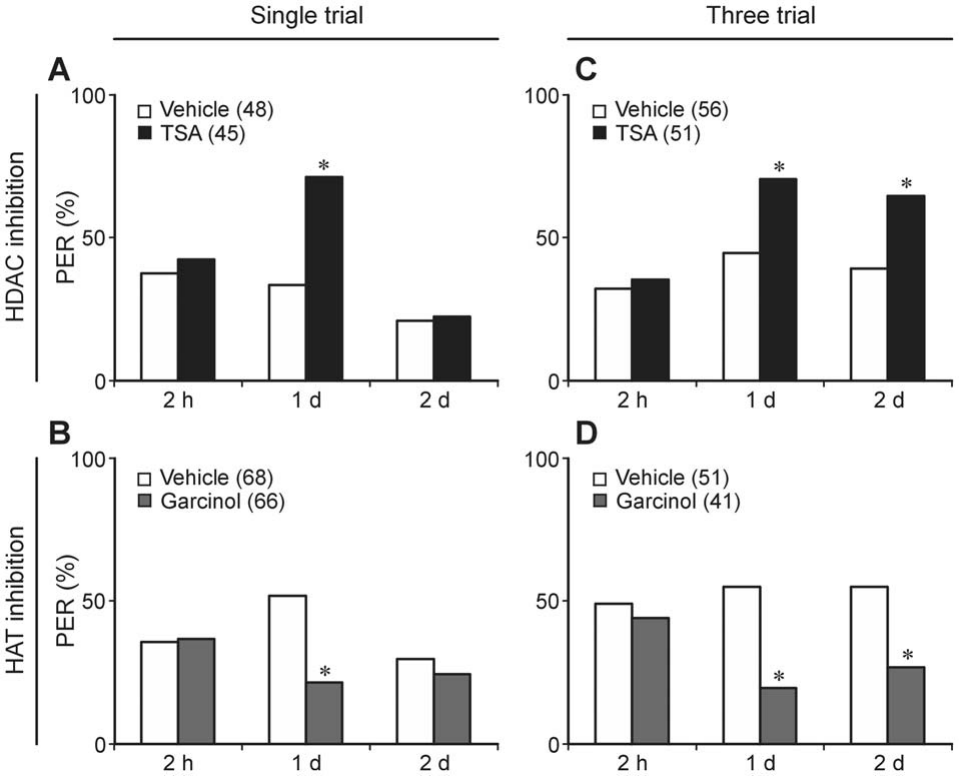
HDAC inhibitor TSA and the HAT inhibitor Garcinol have opposite effects and either enhance or suppress memory after weak or strong training. To interfere with acetylation-dependent processes 2–3 h after conditioning honeybees received a TSA (A, C) or a Garcinol (B, D) injection 0.5 h after weak (single-trial conditioning) (A, B) or strong training (three-trial conditioning) (C, D). In the corresponding control group honeybees were injected with the appropriate vehicle only. The columns show the percentage of animals that elicit the proboscis (PER) during the retrieval test at 2 h, 1 d and 2 d after conditioning. The numbers of animals tested are indicated in brackets behind the different treatments. Chi-square test is used to compare the PERs between the treatments for each tested time point separately. Asterisks indicate significant differences between the groups (details in Results) (Chi-square/Fisher exact test (two-tailed); *p<0.05).

We injected the HAT or HDAC inhibitors 0.5 h after associative olfactory conditioning to observe effects on conditioning-induced acetylation processes 2–3 h after training and the according memory (Fig. 2). Elevation of protein acetylation by HDAC inhibition (TSA) after single-trial training enhances memory performance at 1 d (c2 = 4.56, df = 1, p = 0.033) but not at 2 h (c2 = 0.22, df = 1, p = 0.64) and at 2 d (c2 = 0.03, df = 1, p = 1) after training (Fig. 4A). Down-regulation of protein acetylation by the HAT inhibitor Garcinol causes exactly the opposite effect: it impairs memory at 1 d (c2 = 13.2, df = 1, p = 0.0004) but not at 2 h (c2 = 0.02, df = 1, p = 1) and at 2 d (c2 = 0.46, df = 1, p = 0.56) after single-trial conditioning (Fig. 4B).

Unlike weak training, the interference with acetylation-dependent processes after three-trial conditioning (acquisition see Table 4) affects memory at day 1 and day 2 as well (Fig. 4 C, D). While HDAC inhibition by TSA enhances memory (1d: c2 = 7.33, df = 1, p = 0.007; 2d: c2 = 6.9, df = 1, p = 0.009), HAT inhibition by Garcinol suppresses memory at 1 d and 2 d after training (1d: c2 = 11.9, df = 1, p = 0.0011; 2d: c2 = 7.33, df = 1, p = 0.007). As in case of weak training, memory tested 2 h after three-trial training is neither affected by TSA (Fig. 4 C; 2 h: c2 = 0.12, df = 1, p = 0.73) nor by Garcinol (Fig. 4 D; 2 h: c2 = 0.24, df = 1, p = 0.62).

**Table 4:** Acquisition phase of the three-trial conditioning experiments (Fig. 4) prior to the TSA or Garcinol injection.

Acquisition	HDAC inhibitor		HAT inhibitor	
PER	Vehicle (56)	TSA (51)	Vehicle (51)	Garcinol (41)
**1. trial**	0%	0%	0%	0%
**2. trial**	14%	18%	59%	63%
**3. trial**	23%	18%	67%	71%

The values show the percentage of animals that elicit the proboscis (PER) to the CS of the three successive conditioning trials (CS/US pairings) given with an inter-trial interval of 2 minutes. Pairwise comparison of the 2nd and 3rd trial by the Chi-square/Fisher exact test reveals no differences between TSA/vehicle or Garcinol/vehicle (all p values>0.6).

Thus, after associative conditioning, acetylation-dependent processes act as bidirectional regulators of distinct long-lasting memories in the range of days without affecting mid-term memory in the range of hours.

## Discussion

We demonstrate that in contrast to strong appetitive associative training, weak training leads to a delayed reduction in acetylation of H3K18 in the honeybee brain. Disregarding training strength, the reduction of protein acetylation levels suppresses memory performance. These findings support the “molecular brake pad hypothesis” [8] that proposes a role of learning induced acetylation-dependent processes in memory suppression *in vivo*. The latter hypothesis states that HDACs and associated enzymes act as suppressors - “brake pads” – of gene expression, which can be removed by strong inputs (e.g. three-trial conditioning) to facilitate and modulate gene expression. Our observations that i) strong training causes an elevation in acetylation levels and that ii) elevation of acetylation levels (“release of brake pads”) leads to an improved memory performance are in accordance to findings in other species and support the theorem of a conserved role of acetylation processes in memory formation [7], [9], [23]. As a new aspect we show that weak training (single-trial conditioning) induces biphasic changes in acetylation levels: first (30 min) an elevation that theoretically supports gene expression, followed (≈2–3 h) by a reduction in acetylation levels that might further suppress gene expression in this time window after training. This together with the finding that manipulation of acetylation levels also affects a distinct memory phase (1 d) after weak training supports the idea that acetylation-dependent processes are general modulators of memory formation.

The observation that appetitive associative conditioning changes the dynamic of H3K18ac, while H3K9ac is unaffected in the same samples, argues for an activation of particular HATs (or inactivation of HDACs) by associative learning. This hypothesis is supported by a recent work on the substrate specificity of the CBP (CREB binding protein) intrinsic HAT activity [28]. Deletion of CBP/p300 in cells specifically reduces H3K18 acetylation without affecting H3K9 acetylation suggesting that the honeybee CBP homologue acts as a potential mediator of the learning-induced changes in H3K18 acetylation *in vivo*. However, a learning-induced regulation of distinct HDACs is also feasible. A complex stimulus-induced activation of the acetylation machinery has also been demonstrated in a study on isolated *Aplysia* neurons [13]. Here, different stimuli (5HT and FMRF) trigger distinct acetylation events, which finally result in either activation or suppression of synaptic plasticity. The different acetylation-mediated processes seem to occur in a timely coordinated fashion.

As shown in different species, associative learning leads to an elevation in histone acetylation levels in the contributing neuronal circuits [19], [21], [42]. So far, measurements at single time points after conditioning suggested a positive correlation between the level of histone acetylation and training strength [18], [43]. The detailed monitoring of learning-induced changes in histone acetylation now adds new information with regard to the differences between weak and strong conditioning and - to our knowledge - provides the first evidence of a delayed learning-induced reduction in histone acetylation *in vivo*. Together with the observation that reduced acetylation levels decrease memory performance, our results suggest a contribution of acetylation-mediated processes to memory suppression.

In our current understanding elevation or reduction of histone acetylation modulates memory by improving or impeding transcription processes [8]. In this publication we present first evidence that changes in acetylation also improve and suppress the formation of translation- and transcription-independent memory in honeybees [35], [36]. Although the mechanisms are not investigated yet, it is tempting to speculate that the intrinsic HAT activity of CBP may also contribute to the changes in protein acetylation induced by weak training. The latter scenario is possible, since recent reports demonstrate that CREB and CBP are also involved in the formation of short-term memory [44], [45]. So far CREB and CBP have only been implicated in LTM formation [6], [46]. Complete conditional knockout of CBP in the forebrain of mice causes deficits in formation of LTM but also short-term memory [44]. In line with this, mutant mice with an up-regulated CREB activity in the forebrain enhance short-term memory in addition to LTM [45]. Thus, both studies provide clear evidence that major components regulating transcriptional processes also affect short-term memory. In mice the CBP effect on memory formation is not rescued by elevating acetylation levels. This points to either a very specific action of the CBP's intrinsic HAT activity on targets implicated in transcription regulation, or processes that regulate cellular processes not related to transcription.

Although a detailed analysis does not exist so far, studies in mice provide first evidence that TSA and Garcinol treatments affect different target genes [41], [47]. In hippocampal neurons, TSA treatment causes a time-dependent increase in the levels of *Hdac1* mRNA and HDAC1 protein [47]. In contrast, Garcinol does not affect HDAC1 but changes expression of HDAC2 in hippocampal neurons [41]. So far, only HDAC2 but not HDAC1 has been shown to impair synaptic plasticity and memory formation [20]. Thus, TSA and Garcinol can lead to very specific effects by activating or suppressing distinct genes. The DNA-intercalator actinomycin-D obviously contrasts this pattern by its general or broadband interference with transcription processes. Consequently, TSA/Garcinol on one hand and actinomycin-D on the other hand cause qualitatively different effects on transcription processes underlying memory formation.

In this context it is important to point out that HATs and HDACs also act on non-histone targets that regulate a variety of cellular processes, such as protein turnover [48], [49]. In this regard, the honeybee can provide an ideal system to identify and characterize the targets of the acetylation-dependent regulation of memory formation and to identify the mechanisms contributing to the suppression of short-term memory.

## Materials and Methods

### Materials

Anti-Histone H3 antibody (cat#H0164), anti-rabbit IgG alkaline phosphatase-labeled antibody (cat#A3687), anti-rabbit IgG peroxidase-labeled antibody (cat#A6154), and Trichostatin A (TSA) were from Sigma-Aldrich (Munich, Germany). Anti-acetyl histone H3 (K18) antibody (cat#9675), anti-acetyl histone H3 (K9) antibody (cat#9671), and anti-acetyl lysine antibody (cat#9441) were from Cell Signalling (Frankfurt, Germany). Garcinol was from Biomol (Hamburg, Germany).

### Behavioral analysis

Honeybees (*Apis mellifera carnica*) were caught when leaving the hives for foraging. For gustatory responsiveness tests, and non-associative learning honeybees were caught at the day of the experiment. The next day the drugs were injected 1–2 h prior to the behavioral test as indicated in Results. The responsiveness to appetitive stimuli was measured by stimulating the antennae with gradually increasing sucrose concentrations (0 M, 30 mM, and 1 M) at an inter-stimulus interval of 2 min. The proboscis extension response (PER) was monitored for each stimulus. Sensitization was measured by testing the increased response (PER) probability to an odor stimulus (clove oil), applied 15 s after an arousing appetitive stimulus (1 M sucrose) to the antennae of hungry bees. Animals showing no PER to the sensitizing 1 M sucrose stimulus were excluded from the experiment (<3%). Habituation was tested by repeated stimulation (0.5 s inter-stimulus interval) of an antenna with 1 M sucrose solution. The number of elicited PERs until 5 consecutive PER failures is defined as the habituation criterion. Animals that were not habituated after 50 stimuli were excluded from the analysis (<4%). The dishabituating stimulus, a sucrose stimulus that follows the 5 consecutive failures, was applied to the contra-lateral antenna. Only animals showing a PER to the dishabituating stimulus were included in the analysis (>95%).

For appetitive olfactory conditioning animals were caught the day before training. An associative olfactory conditioning trial consisted of pairing an odor stimulus (conditioned stimulus, CS; clove oil for 5 s) with an appetitive reward stimulus (unconditioned stimulus, US; 1 M sucrose for 4 s) [50]. In case of the unpaired stimulation, the honeybees received a US stimulus followed by CS stimulation 15 s later. An exhaust behind the animals removed lingering odor. Three seconds after CS onset, the unconditioned stimulus (US) was presented by touching both antennae and after extension of the proboscis the animals were allowed to lick sucrose solution for 3 s. Animals received either one or three successive conditioning trials with an inter-trial interval of 2 min. The retention tests were performed 2 h, 1 d and 2 d after the training by presenting the CS alone. Animals not responding to the US during conditioning were excluded from the experiment (<2%). The behavioral experiments were performed in the years 2008 (April–October) and 2009 (February–September) in Saarbrücken, Germany. To avoid hive-dependent effects, the bees were collected from at least 3 different hives. These animals were pre-experimentally mixed and randomly assigned to the separate experimental groups. After this pre-experimental group-assignment no changes were made.

### Drug application

Drugs were injected into the hemolymph of the thorax (1 µl volume each) at times as indicated in therespective results. For injection we used a calibrated glass capillary inserted through a hole pricked into the tergite. Following solutions were used: Garcinol, 6 mM in 100% DMSO; Trichostatin A (TSA), 1.65 mM in PBS [137 mM NaCl; 2.7 mM KCl; 10.1 mM Na_2_HPO_4_; 1.8 mM KH_2_PO_4_] containing 20% DMSO; Act.D, 1.8 mM in PBS containing 20% DMSO. The corresponding control groups were injected with the appropriate vehicle only. At an average bodyweight of 100 mg the substance concentrations per bee were: Garcinol, 60 µM; TSA, 16 µM and Act.D, 18 µM.

### Antibody specification by Western blot

The specificity of anti-acetyl histone H3 (K18), anti-acetyl histone H3 (K9), anti-histone H3, and anti-acetyl lysine antibodies for honeybee brain was tested by Western blot analysis. A freshly dissected brain was immediately homogenized in 200 µl homogenization buffer (PBS, containing 1 mM EDTA and 5 mM sodium butyrate). After adding 40 µl SDS-sample buffer (0.5 M Tris-HCl, pH 6.8, containing 5% SDS, 5% 2-mercaptoethanol, and 20% glycerol) the sample was incubated at 95°C for 5 min in a thermo block and loaded on a SDS-polyacrylamide (stacking gel 4%, separation gel 15%). After separation the proteins were transferred to a nitrocellulose membrane using a semi-dry transfer method. After blocking (1 h in PBS; 0.5% BSA; 0.1% Tween 20) the membrane was cut into stripes, each incubated with a different primary antibody (anti-histone H3 1∶5000; anti-acetyl histone H3 (K18) 1∶1000; anti-acetyl histone H3 (K9) 1∶1000; anti-acetyl lysine 1∶2000; all diluted in PBS containing 0.5% BSA; 0.1% Tween 20). After rinsing and washing (3×5 min each), the stripes were incubated for 1 h with the secondary antibody (peroxidase-labeled anti-rabbit IgG) (1∶10000 in PBS; 0.5% BSA; 0.1% Tween 20), washed again (3×5 min each), and developed using chemiluminescence detection (ECL, PerkinElmer Inc., Waltham, MA USA).

### Immunohistochemistry

Brains were dissected and fixed in Carnoy (30 ml ethanol abs., 15 ml chloroform, 5 ml glacial acetic acid) for 4 hours at room temperature. For paraffin sections, the tissue was dehydrated in increasing grades of ethanol terminating in 100% dehydrated ethanol, followed by isopropanol and embedded in paraplast (Sigma, St. Louis, MO). Sections (7 µM) were mounted on poly-D-lysine coated slides. After rehydration slides were washed (2×5 min) with PBS-T (PBS, containing 0.1% Triton X-100) and incubated in blocking solution (PBS-T containing 0.5% BSA) for 1 hour at room temperature. Antibodies against H3, acetylated H3K9 and H3K18 were used as primary antibodies. Using dilutions of 1∶1000 in blocking solution of either antibody, the sections were incubated overnight at 4°C. After washing with PBS-T (3×5 min) the sections were incubated for 1.5 hours at room temperature with Cy3-labeled anti-rabbit IgG) (1∶2000 in blocking solution). After washing with PBS-T (3×5 min) the sections were mounted with 50% glycerol.

### Quantification of protein acetylation

We used the enzyme-linked immunosorbent assay (ELISA) to quantify H3K18ac, H3K9ac, acetylated proteins, and H3 in each of the brain samples. At the indicated times after olfactory conditioning or after drug injection, honeybees were shortly cooled on ice, the heads were cut off, and mounted on wax. Within 30 s the cuticle was opened, the central brain with the mushroom bodies dissected, and homogenized in 500 µl homogenization buffer (PBS containing 1 mM EDTA and 5 mM sodium butyrate). Each of the samples were transferred to four micro titer plates, one for each antigen (F96 Maxisorp, NUNC-IMMUNO, Langenselbold, Germany) (50 µl each) and diluted in five consecutive steps (1∶2) with homogenization buffer. After 1 h incubation, the wells were blocked for 1 h with blocking buffer (PBS containing 0.5% BSA), and the different primary antibodies [anti-acetyl histone H3 (K18) (1∶1000), anti-acetyl histone H3 (K9) (1∶1000), anti-histone H3 (1∶5000); and anti-acetyl lysine (1∶2000); all diluted in PBS containing 0.5% BSA] were applied to the corresponding micro titer plates and incubated overnight at 4°C. After washing, anti-rabbit IgG alkaline phosphatase conjugated antibody (1∶4000 in PBS containing 0.5% BSA) was applied and incubated for 1 h at room temperature. After adding the phosphatase substrate solution (1 mM 4-nitrophenylphosphate disodium salt (p-NPP) in 0.1 M Tris/HCl pH 8,7; 1 mM MgCl_2_) the conversion of the substrate was quantified by a plate reader (safire^2^, Tecan, Crailsheim, Germany) at 405 nm using 600 nm as background.

The ELISA data were evaluated as described previously [51]. The samples of the different groups within one experiment were placed on the same ELISA plates (separate copy plates for each antibody). The slope calculated from the optical density values of the dilution steps (linear range) represented the relative amount of antigen in a given sample. To compensate for differences in the staining procedure of the different plates (several plates were required to test the indicated numbers of samples) the calculated slope of each sample on a plate was normalized to the average slope of all the samples on this plate. The means of the relative amounts of H3 did not differ between the groups (Table 1, 2). Thus, in each sample the normalized H3, H3K18ac, and H3K9ac values were used to calculate the H3K18ac/H3 or H3K9ac/H3 for each of the samples. After normalization to the corresponding control ratio of a given experiment, the mean ± SD was calculated. The Two-tailed Student's test was used to compare the data.

### Statistical analysis

SYSTAT10 was used for the statistical analysis. The relative amounts of H3 after different training procedures and after injection of TSA or Garcinol were tested with ANOVA (Table 1, 2). The Student's t-test (two-tailed) was used to compare the relative acetylation levels at the different time points after training or after injection of TSA or Garcinol (Fig. 2 and 3). The habituation data were compared with Student's t-test and responsiveness and sensitization with Chi-Square/Fisher exact test. We use the conservative Chi-square/Fisher exact test for pairwise comparisons of the treatment at distinct time points. We do not intend to compare between the different time points by ANOVArm [52], until the transient phasic effects after single-trial conditioning (due to the non-continuous characteristics the variances strongly depend on the selected time points) and its temporal interacting with the non-affected processes are characterized. Moreover due to the very low scores in some of the “cells” the application of ANOVArm is not without problems. In all cases p<0.05 is considered as significant.
